# Genome sequence of bacteriophage Aoka, a cluster FO phage isolated using *Arthrobacter globiformis*

**DOI:** 10.1128/MRA.00454-23

**Published:** 2023-09-22

**Authors:** Aidan Sasaoka, Olivia Arellano, Xavier R. Hatch, Amaya M. Garcia Costas

**Affiliations:** 1 Department of Biology, Colorado State University-Pueblo, Pueblo, Colorado, USA; Portland State University, Portland, Oregon, USA

**Keywords:** actinophage, bacteriophage, *Arthrobacter globiformis*, phage genome

## Abstract

We report the discovery and genome sequence of bacteriophage Aoka, an actinobacteriophage isolated from a soil sample in Pueblo, Colorado using *Arthrobacter globiformis*, B2880-SEA. Its genome length is 36,744 base pairs with 54 protein-coding genes. Based on gene content similarity to other actinobacteriophages, Aoka is assigned to cluster FO.

## ANNOUNCEMENT

Bacteriophage are viruses that infect bacteria (1, 2), resulting in bacterial-phage co-evolution (1, 3, 4). Knowledge of bacteriophage gene content and functions is essential to better understand the evolutionary and physiological impacts on bacteria of these infections, yet current findings indicate that the phage genetic space is mostly unknown (5, 6). Here, we present the genome of actinobacteriophage Aoka, sequenced in collaboration with the SEA-PHAGES program sponsored by Howard Hughes Medical Institute (HHMI) (7). Over a third of the open reading frames in Aoka’s genome code for unique proteins of unknown functions as of May 2023.

**Fig 1:**
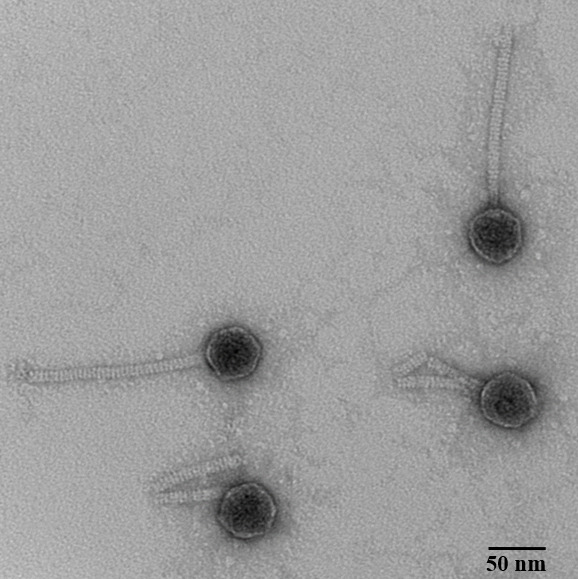
Transmission electron micrographs of phage Aoka. A high-titer (>1.0 × 10^5^ pfu/mL) lysate was negatively stained with 1% uranyl acetate and visualized in a Thermo Fisher Tecnai G^2^ 12 BioTwin electron microscope at 120.0 kV.

Aoka was isolated and purified from soil collected around a tree stump in a grassfield in Pueblo, Colorado (38.346111 N, 104.704722 W) in September 2022 using the host *A. globiformis* B2880-SEA and a host enrichment method with PYCa media at 30°C as outlined in the SEA-PHAGES manual (8), with two rounds of plaque purification. Aoka consistently forms large clear plaques and exhibits a Siphovirus morphology (9) with a capsid 45 ± 5 nm in diameter and tail 140 ± 5 nm in length (*n* = 3; Fig. 1).

Genomic DNA extraction was performed from phage lysate using Promega’s Wizard DNA Extraction Kit (10). A sequencing library was prepared with a NEBNext Ultra II FS kit and sequenced using Illumina MiSeq platform (v3 reagents) yielding 414,384 150-base single-end reads. Raw reads were assembled using Newbler 2.9, with default settings, into a single-phage contig with approximate average coverage of 1,673-fold; the contig was checked for completeness, accuracy, and phage genomic termini with Consed 29 as described previously (10, 11). The genome is 36,744 bp long and has a G + C content of 68.6% with 11-bp 3′ single-stranded overhangs (5′-TTCGCCCGTTA-3′). Based on average nucleotide similarity of at least 35% to phages in the Actinobacteriophage database, Aoka is assigned to cluster FO (12, 13).

An automated annotation was generated using Glimmer (14) and GeneMark (15) and, subsequently, manually curated using DNA Master (16), Phamerator (17), and Starterator (18). Functions for each coding sequence were assigned based on top hits from searches using NCBI BLASTP (19), Phagesdb BLASTP (20), and HHpred (21). Membrane proteins were identified using TMHMM v2.0 (22) and SOSUI (23). Aragorn (24) and tRNAscan-SE (25) were used to identify tRNAs. All tools were run with default parameters.

Aoka is predicted to have 54 protein-coding genes; 24 of which (45%) have assigned functions, and 30 (55%) were annotated as hypothetical proteins. Currently, 19 (35%) of these hypotheticals are unique genes to Aoka (26, 27). No tRNA-coding genes were found. Most genes (*n* = 51) are predicted to be transcribed in a forward orientation and three in reverse orientation (Genes 27 and 32; both helix-turn-helix DNA binding domain, and Gene 28; hypothetical). No repressor or integrase coding genes were found suggesting Aoka is a lytic phage.

A BLASTn search using the nucleotide sequence of the entire Aoka genome as query against the phagesdb.org database (January 2022) returned phage Maja (84% identity; GenBank accession number MK279899) as most similar. Other similar phage retrieved belongs to clusters FB and AS (subcluster AS1) that also contain *Arthrobacter*-infecting phage.

## Data Availability

The complete genome sequence of Aoka has been deposited in GenBank with accession number ON755180, Bioproject accession number PRJNA488469, and SRA accession number SRX14443516.
